# Autophagy–Actin Crosstalk: Implications for Cellular Homeostasis and Disease

**DOI:** 10.3390/cells15080665

**Published:** 2026-04-09

**Authors:** Adelaide Ohui Fierti, Rachel Geneva Rozsa, Anjali Potu, Anam Tajammal, Hui Li

**Affiliations:** Department of Pathology, School of Medicine, University of Virginia, Charlottesville, VA 22903, USA; yur9tm@virginia.edu (A.O.F.); psr2he@virginia.edu (R.G.R.); sca6wk@virginia.edu (A.P.); at7kd@virginia.edu (A.T.)

**Keywords:** autophagy, actin remodeling, actin polymerization, actin depolymerization, cellular homeostasis, actin binding proteins, tumor metastasis, neurodegeneration

## Abstract

Actin is a key component of the cytoskeleton and plays diverse roles in cellular processes. Autophagy regulates homeostasis through various mechanisms that recycle nutrients and degrade unnecessary or harmful cellular components and aggregates. These two processes are engaged in a highly conserved crosstalk through which they regulate each other, including autophagolysosomal formation and regulation of actin dynamics. The regulation of autophagy is involved in cancer, neurodegeneration, infectious diseases, and inflammation, providing possible avenues for treatments for these diseases. In this review, we summarize current knowledge of the actin–autophagy interplay and regulation, and explore the possible implications for disease progression and therapies. Although more research is necessary to strengthen the effectiveness of therapies that target the regulation of autophagy and actin dynamics, significant strides have already been made, clearly indicating the potential benefit of targeting these processes.

## 1. Introduction

### 1.1. Overview of Autophagy: Definition, Mechanisms, and Biological Significance

Autophagy is a highly conserved catabolic process in eukaryotic cells by which cells recycle and degrade their cellular components in the lysosomal pathway. The term derives from the Greek words auto (“self”) and phagy (“eating”), reflecting its role in cellular self-digestion and renewal [1]. Initially described in the 1960s through electron microscopy observations of double-membraned vesicles in hepatocytes [2], autophagy is now recognized as a central regulator of cellular homeostasis, survival, adaptation under stress conditions, and cell death. By eliminating damaged organelles, misfolded proteins, and invading pathogens, autophagy allows cells to maintain cellular homeostasis and integrity [1]. Autophagy is activated in response to a wide range of stressors that disrupt cellular homeostasis including starvation which results from nutrient and energy deprivation [3,4], hypoxia [5], and ER stress which arise from unfolded protein response [6,7]. It is also activated in response to reactive oxygen species [8], mechanical and inflammatory stress [9], damaged organelles such as damaged mitochondria peroxisomes [10,11], infection from pathogens [12], and cytotoxic insults [13]. Beyond damage control, autophagy is essential and activated by physiological processes such as differentiation and development [14,15]. Autophagy activation allows elimination of cargos which are intercellular components that are selectively or non-selectively accumulated for lysosomal degradation and may include damaged organelles, pathogens, protein aggregates, misfolded proteins, lipid droplets, ribosomes, portions of the nucleus, lipid droplets, and excess cytoplasmic content [16]. During periods such as of starvation or metabolic stress, autophagy recycles nutrients to sustain cellular energy production by breaking down unnecessary structures [17]. It eliminates intracellular pathogens in a process called xenophagy and acts as a tumor-suppressive mechanism by reducing oxidative stress and genomic instability, while also supporting proper development and differentiation [18,19]. In the nervous system, autophagy is crucial for preventing the buildup of protein aggregates associated with neurodegenerative diseases such as Alzheimer’s, Huntington’s, and Parkinson’s [20,21,22].

There are three major types of autophagy. (1) Macroautophagy involves the canonical autophagy pathway where a phagophore forms, elongates and engulfs cargo, forms the autophagosome, and fuses with a lysosome to form the autolysosome for degradation. Cargos in macroautophagy include organelles, pathogens, aggregated proteins, lipids. Macroautophagy mediates bulk degradation and recycling of cytoplasmic materials [23]. (2) Microautophagy, unlike macroautophagy, involves the lysosomes invagination or protrusion and engulfing cytoplasmic content and fusion with late endosomes to form endolysosomes. Cargos may include lipid droplets, proteins, and organelles. The primary function is to promote membrane biogenesis and basal turnover to maintain cellular homeostasis [24]. Chaperone-mediated autophagy (CMA) is a selective form of autophagy where the chaperone Hsc70 recognizes and binds to specific soluble proteins with KFERQ-like motif to form a complex. The complex is then recruited to the lysosome by the LAMP-2A lysosomal receptor. The proteins unfold and translocate across the lysosomal membrane for degradation [25]. This process is important for stress response, protein quality control, and regulation of metabolism. This review will focus on macroautophagy and its cellular dynamics.

**Figure 1:**
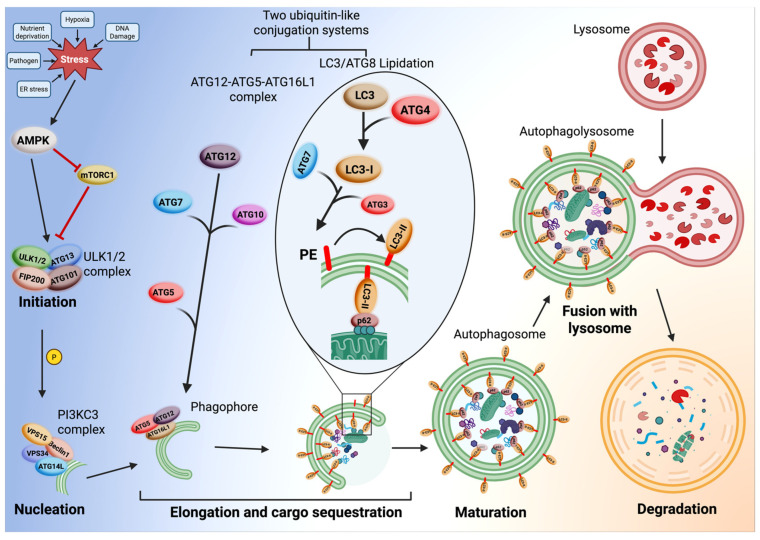
The macroautophagy pathway proceeds through the following steps: Initiation, nucleation, elongation, maturation, fusion, and degradation. Created in BioRender. Fierti, A. (2026) https://BioRender.com/e82l5he accessed on 12 February 2026.

Mechanistically, macroautophagy proceeds through several coordinated steps: initiation, nucleation, elongation, maturation, fusion, and degradation (Figure 1). The initiation step is triggered by stress signals including nutrient deprivation, hypoxia, damaged organelles, protein aggregation, etc. Initiation is regulated by the interplay between mTORC1 and AMPK. mTORC1 acts as a negative regulator of autophagy; when nutrients and growth factors are abundant, it remains active and inhibits autophagy, promoting cell growth and anabolic processes [26]. Under starvation or stress, mTORC1 activity is suppressed while AMP-activated protein kinase (AMPK) is activated [27]. AMPK promotes initiation simultaneously by (i) direct phosphorylation of Unc-51-like autophagy activating kinase 1 (ULK1) and (ii) inhibition of mTORC1. This dual action effectively activates the ULK1 complex (comprised of ULK1, ATG13, FIP200, and ATG101), initiating autophagy [28].

This is followed by nucleation, where the phagophore, a small and isolated double membrane structure that buds from the endoplasmic reticulum (ER), forms a cup-shaped structure. A key target of the activated ULK1 complex is the Class III Phosphatidylinositol 3-kinase Complex I (PI3KC3-C1). This complex contains the lipid kinase VPS34 and the regulatory protein Beclin1, as well as VPS15 and ATG14L. When activated, it produces phosphatidylinositol 3-phosphate (PI3P) on the surface of the growing phagophore. This PI3P acts as a critical signal, creating a specialized membrane domain that serves as a docking site for downstream ATG proteins (autophagy-related proteins) required for the elongation step of autophagy [29,30,31].

The elongation step involves phagophore expansion and cargo sequestration. The phagophore engulfs cytoplasmic components or cargo such as proteins, lipids, and organelles. This process is driven by the coordinated action of two ubiquitin-like conjugation systems [32,33]. (i) The ATG12-ATG5-ATG16L1 Complex begins with the activation of the ubiquitin-like protein ATG12. The E1-like enzyme ATG7 activates ATG12 by covalently binding to it. ATG7 then transfers ATG12 to the E2-like enzyme ATG10. ATG10 facilitates the conjugation of ATG12 to ATG5, forming a stable ATG12-ATG5 conjugate. This conjugate then binds to the scaffolding protein ATG16L1 to form a large, multimeric ATG12- ATG5-ATG16L1 complex [34,35]. This complex localizes to the phagophore membrane and is thought to act as an E3-like ligase for the LC3/ATG8 Lipidation System. (ii) The LC3/ATG8 Lipidation System creates the active form called LC3-I. The cytosolic protein pro-LC3 (homolog of ATG8) is processed by the cysteine protease ATG4 to expose a glycine residue, creating the active form called LC3-I [36]. The E1-like ATG7 activates LC3-I and also transfers LC3- I to E2-like enzyme ATG3 [37,38]. The ATG12-ATG5-ATG16L1 complex, which acts as E3-like ligase, allows conjugation of LC3-I from ATG3 to the lipid phosphatidylethanolamine (PE), embedded in the phagophore membrane, creating LC3-II, which is now covalently anchored to both the inner and outer membranes of the growing phagophore [39]. LC3-II is essential for membrane expansion and cargo selection via receptors such as p62/SQSTM1 or NBR1 [40,41].

Maturation involves autophagosome completion, where the edges of the phagophore close to form a double-membrane autophagosome. This is followed by the fusion of the autophagosome with a lysosome to form autolysosomes, mediated by SNARE proteins [42]. Autophagosome fuses with lysosomes using the HOPS complex for tethering, Rab GTPases (Rab7) which regulate fusion, and the SNARE proteins which mediate membrane fusion, to form the autophagolysosome [43].

After fusion, the lysosomal hydrolases made up of proteases, lipases, and nucleases degrade the cargo (such as aggregated proteins, lipids, organelles) into amino acids, fatty acids, and nucleic acids for reuse. The autophagolysosome is now capable of performing the degradation step, which allows it to recycle cellular contents and destroy potentially harmful protein aggregates. This process ensures cellular homeostasis under stress and starvation conditions [44].

Autophagy is essential for cell function and survival. It helps maintain internal homeostasis by degrading damaged mitochondria and toxic protein aggregates [45]. Additionally, enhanced autophagy has been linked to increased lifespan in various organisms, making it a target of interest in aging and longevity research. Overall, autophagy is a vital process for managing health and disease, and its dysfunction is associated with a wide range of conditions, from cancer to neurodegeneration [17].

### 1.2. Overview of Actin Dynamics: Structure, Regulation, and Functions

Actin is the most abundant protein in most eukaryotic cells. It participates in more interprotein interactions than any known protein and plays a fundamental role in maintaining cell shape and polarity, enabling movement, regulating transcription, and facilitating intracellular transport [46]. It exists in two main forms: monomeric globular actin (G-actin) and filamentous actin (F-actin), the latter of which polymerizes from G-actin subunits in a dynamic, reversible process [47]. The polymerization of G-actin into F-actin is polarized, with a fast-growing, positive “barbed” end and a slower-growing, negative “pointed” end that allows for directional growth and shrinkage in response to cellular signals. The process is highly regulated and energy dependent. It begins with nucleation, facilitated by proteins such as AVIL and the Arp2/3 complex, which initiates branched filament networks, and formins which promote linear filament nucleation and elongation [48,49]. Profilin promotes actin filament elongation by catalyzing the exchange of ADP for ATP on G-actin and delivering it to the barbed ends, while also capping proteins to stabilize or limit filament growth [50]. Depolymerization is equally regulated by severing proteins like cofilin, which promote the disassembly and recycling of actin monomers [51]. Thymosin-β4, another key regulator, sequesters G-actin to buffer the pool of polymerizable monomers. Actin binding proteins (ABPs) control filament dynamics by influencing polymerization, branching, severing, and crosslinking, making the actin cytoskeleton a highly adaptable network [52].

The dynamic remodeling of actin filaments underlies numerous essential cellular processes. In motile cells, branched actin networks drive lamellipodia formation for directional migration, whereas bundled filaments form filopodia that probe the extracellular environment [53]. Actin filaments are crucial for intracellular trafficking, endocytosis, cytokinesis, and maintaining cell shape and mechanical integrity [54]. In epithelial cells, actin supports apical structures such as microvilli and adherens junctions, while in neurons, actin-rich growth cones guide axon pathfinding [55]. Beyond structural roles, actin dynamics influence nuclear processes, including chromatin remodeling and transcription regulation, and integrate with signaling pathways to allow cells to respond and adapt to environmental cues and mechanical stress [56,57]. In human cell lines, the actin cytoskeleton is directly involved in changes in the number of autophagosomes [57], which allows for a transient autophagy response that helps the cell adapt to new conditions [56]. Dysregulation of actin dynamics has been implicated in a wide range of diseases, including cancer metastasis, immune dysfunction, and neurodegeneration, underscoring the essential role of actin in both cellular function and organismal health [58].

### 1.3. Rational for Studying Actin–Autophagy Crosstalk

The actin cytoskeleton and autophagy are both highly regulated; autophagy is a dynamic degradative process, and the actin cytoskeleton is a dynamic structural network. Both are essential for maintaining cellular homeostasis [59]. Emerging evidence suggests that these systems are functionally interconnected: remodeling of the actin cytoskeleton facilitates key stages of autophagy, including autophagosome formation, trafficking, and fusion with lysosomes, while autophagy can also influence actin dynamics by degrading specific actin-regulatory proteins [60]. Studying the crosstalk between these processes provides insight into how cells coordinate structural organization with degradation and recycling pathways, particularly under conditions of stress or rapid adaptation [59]. Understanding this interplay can reveal how cells fine-tune their behavior and preserve homeostatic balance during processes such as migration, division, or differentiation.

### 1.4. Significance in Health and Disease

Disruption in the balance between autophagy and actin dynamics has been linked to a variety of human diseases [59]. In cancer, abnormal actin remodeling and impaired autophagy contribute to uncontrolled cell growth, tumor progression, metastasis, and resistance to therapy [61]. In neurodegenerative diseases such as Alzheimer’s and Parkinson’s disease, defective autophagy results in the accumulation of toxic protein aggregates, while dysregulated actin dynamics disrupt neuronal transport and synaptic function [62]. In many infectious diseases, pathogens exploit both the autophagy pathway and the actin cytoskeleton to evade immune detection and facilitate intracellular spread [63]. Understanding how these systems interact could uncover novel therapeutic targets and improve our ability to offer therapeutic opportunities in diseases characterized by cellular disorganization and defective clearance mechanisms.

## 2. Molecular Mechanisms: Autophagy and Actin Remodeling Interdependency

### 2.1. Autophagy-Regulated Actin Remodeling

Many autophagy-related proteins (Atg/ATG) interact with actin-related proteins (Arps) to carry out their functions, and vice versa. Arp2 fulfills the role of linking autophagic machinery with the actin cytoskeleton by colocalizing with ATG9. This regulates the dynamics of ATG9 movement and transport [64]. During autophagy, ATG9 vesicles transport membrane components from endosomes to autophagosomes, an activity that is dependent on assembly of actin cytoskeleton on ATG9A vesicles [65]. ATG9 is then integrated and diluted in the autophagosome membrane as it expands during development [66]. In liver sinusoidal endothelial cells, autophagy, degradation of structural protein caveolin-1 (Cav-1), and F-actin remodeling are triggered together during certain processes, including CCl4-induced defenestration of the cells. During this process, autophagic degradation of Cav-1 inhibits the NO-dependent pathway and causes F-actin remodeling, which promotes defenestration. The interplay between these proteins has been confirmed by observing that the presence of autophagy inhibitor 3MA led to decreased degradation of Cav-1 and increased F-actin remodeling. Autophagy inhibition can also occur using bafilomycin or ATG5-siRNA [67]. In the reverse direction, overexpression of Cav-1 counters some of the effects of autophagic degradation [67].

As a result of the interplay between autophagy and actin remodeling, autophagy plays a role in several actin-based cellular processes. Gamete generation of budding yeast occurs via collapse of a subset of the cortical endoplasmic reticulum during anaphase II, which separates into a spatially distinct compartment. This programmed collapse is controlled in part by the actin cytoskeleton [68]. Actin remodeling of the endoplasmic reticulum is coupled to its degradation by selective autophagy, which is regulated by temporally specific expression of Atg40 [68]. In other phagocytic cells, cell-to-cell transmission of pathogenic mycobacteria must occur via nonlytic ejection through the ejectosome, which is composed primarily of F-actin. During the ejection process, autophagic machinery maintains the integrity of the host cell’s plasma membrane, as the mycobacteria being ejected are carried by distinct polar autophagocytic vacuoles [69]. In the absence of this mechanism, transmission is inhibited and the host cell’s plasma membrane becomes compromised, leading to cell death [69].

### 2.2. Actin Cytoskeleton’s Role in Autophagy Regulation

**Figure 2:**
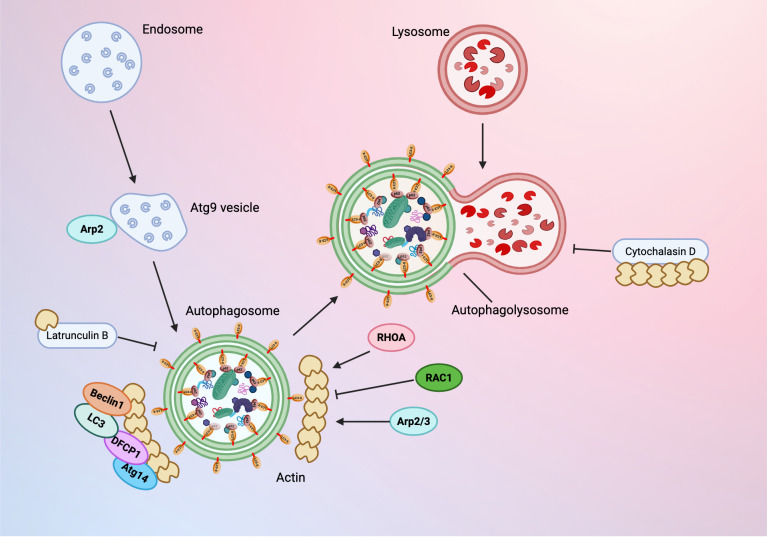
Compilation of autophagy regulation mechanisms by actin-related proteins (Arps), autophagy-related proteins (Atg/ATGs), and other regulatory proteins. Created in BioRender. Fierti, A. (2026) https://BioRender.com/ft2zfg7 accessed on 12 February 2026.

The most common form of autophagy is stimulated by nutrient deprivation, which leads to the increase of autophagosomes in the cell. Actin is critical for this process; depolymerization of the actin cytoskeleton inhibits new production of autophagic vesicles in response to starvation stimulus, although maturation of any remaining autophagosomes is not affected. Actin is primarily involved during the early stages of autophagosome formation and is linked to the generation of PtdIns3P [70]. During this initial membrane remodeling stage, actin function is regulated by Rho proteins, RHOA and RAC1. Within the context of starvation-mediated autophagy, RHOA activates the autophagy process and RAC1 is inhibiting [70] as shown in Figure 2. RHOA signaling also mediates mechanically induced autophagy, highlighting its larger role in autophagosome formation beyond starvation conditions.

Actin polymerization and branching produce mechanical forces associated with membrane deformation during autophagy, as well as endocytosis and phagocytosis. During starvation-mediated autophagy, F-actin colocalizes with key degradation and autophagosome formation proteins Atg14, DFCP1, Beclin1, and LC3 [65]. Actin networks are assembled on both sides of the phagosome to stabilize the compartment and promote membrane curvature. Actin is hypothesized to either act as a scaffold or generate propulsion forces to maintain the high curvature required for phagophore expansion after the omegasome is formed. Arp2/3 inhibition interferes with branched actin network formation and prevents bending of the phagophore, leading to omegasome collapse. The comet tail mechanism of branched actin networks transports the mature autophagosome through the cytoplasm. The actin cytoskeleton then supports fusion of autophagosomes and lysosomes to form the autophagolysosome [65]. Treatment with actin-depolymerizing drugs inhibits autophagosome development; Latrunculin B inhibits starvation-stimulated autophagosome production and Cytochalasin D inhibits autophagosome maturation [65]. Both drugs act through inhibition of actin polymerization; Latrunculin B and the related Latrunculin A bind to G-actin monomers, forming complexes that inhibit polymerization. Latrunculin B is capable of disrupting the actin cytoskeleton at concentrations comparable to the actin monomer binding constant (0.2 μM) [71]. In contrast, Cytochalasin D effectively disrupts F-actin at concentrations 1000 times higher than its dissociation constant. Cytochalasin D may compete with βCAP73 capping proteins that aid the ERM protein subfamily in linking the barbed ends of F-actin to the plasma membrane; competition by Cytochalasin D introduces mechanical changes that disrupt this activity and perturb the actin cytoskeleton [71]. While actin is present during the majority of mammalian autophagosome formation as a driving force and structural support, it is not detected at relevant endoplasmic reticulum sites during autophagy initiation [65]. Notably, actin filaments are not necessary for autophagy in plants; basal autophagy and upregulation of nocturnal autophagy and salt stress-induced autophagy occur independently of actin disruption in Nicotiana benthamiana and Arabidopsis [72].

The actin cytoskeleton interacts with multiple myosins to promote autophagy. Non-muscle myosin IIA delivers the membrane for initial autophagosome formation, while myosin IC and myosin VI provide membranes for autophagosome maturation and final autophagosome–lysosome fusion [73].

While starvation-mediated autophagy is nonselective and provides a nonselective means of recycling energy from less-necessary macromolecules, autophagy occurs independently of starvation with quality control (QC) autophagy. QC autophagy involves the selective destruction of protein aggregates and damaged organelles that may cause harm to the cell and is established by ubiquitin-binding deacetylase HDAC6 [74]. HDAC6 promotes F-actin remodeling, which stimulates autophagosome–lysosome fusion. Cortactin promotes actin polymerization and rearrangement during QC autophagy, but HDAC6 and cortactin are not necessary components of starvation-mediated autophagy [74].

Autophagy is regulated at a transcriptional level by inositol-requiring mutant 80 (INO80) and histone variant H2A.Z. INO80 is composed of multiple subunits containing actin and several actin-related proteins (Arp4, Arp5, and Arp8) to help INO80 recognize and bind DNA and nucleosome structures [75]. In its stable form, INO80 evicts H2A.Z from autophagy-related genes, causing transcriptional repression of those genes and repression of autophagy. In parallel, histone deacetylase Rpd3L complex deacetylates H2A.Z and reduces its association with autophagy-related gene promoters, repressing transcription. INO80 is protected from autophagic degradation by deacetylation at lysine 929 by Rpd3L complex [75]. These steps occur to repress autophagy under nutrient-rich environmental conditions. However, nitrogen starvation induces inactivation of the TORC1 complex, leading to reduced Rpd3L activity. INO80 and H2A.Z subsequently undergo increased acetylation, and autophagy-related genes are transcribed [75]. This autophagic flux is necessary for the health and maintenance of the cell. Decreased autophagic flux is associated with cancer and has been shown to cause increases in pathological symptoms of chronic diseases such as heart disease and dementia hallmarks. Because physiological nutrient conditions affect the repression of autophagy-related genes, targeting biochemical signaling related to levels of nutrition may effectively increase autophagic flux [76].

### 2.3. Actin Binding Proteins and Actin Dynamics as Regulatory Brakes on Autophagic Flux

**Figure 3:**
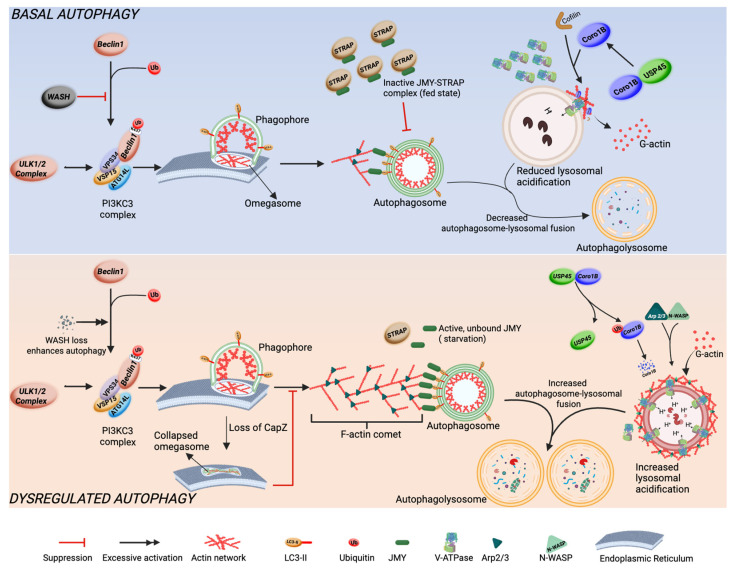
Actin binding proteins and actin dynamics as regulatory breaks on autophagic flux. Dysregulated autophagy describes non-basal conditions in which autophagy is induced, excessive, suppressed, or inhibited Created in BioRender. Fierti, A. (2026) https://BioRender.com/zatymvx accessed on 12 February 2026.

The tight regulation of actin cytoskeleton is crucial for the maintenance of autophagic flux under basal conditions [59]. While some actin binding proteins remodel the actin cytoskeleton to positively promote autophagic flux, other actin binding proteins are negative regulators, acting as a brake to ensure steady and proper progression of the autophagy pathway. As a result, loss of these negative regulators may exaggerate or inhibit autophagic flux (Figure 3).

WASH (Wiskott–Aldrich syndrome protein and SCAR homologue) functions as a negative regulator of autophagy in mammalian cells. While primarily recognized for its role in activating Arp2/3 complex to promote the formation branched actin network on endosomes, WASH also influences autophagy by interacting with the autophagy-related protein Beclin 1 [77]. Under normal circumstances, Beclin 1 undergoes ubiquitination at lysine 437 by the E3 ligase Ambra1, which enhances its interaction with Vps34 kinase and facilitates autophagy. WASH obstructs this mechanism by inhibiting Beclin1 ubiquitination, thus diminishing Vps34 activity and hindering the initiation of autophagy. As a result, a lack of WASH leads to excessive autophagy and contributes to early embryonic lethality, emphasizing its essential role in sustaining cellular homeostasis by suppressing autophagy [78].

Another actin binding protein known to act indirectly as a brake on autophagy is JMY (Junction mediating and regulatory protein). As a factor for activating the Arp2/3-dependent actin assembly [79], it has three WASP homology 2 (WH2) domains for interaction with G-actin, capable of nucleating actin assembly independent of Arp2/3 [80], and LC3-interaction Region (LIR) for interaction with LC3 during autophagy [81]. Under conditions of starvation, LC3 binds to the LIR region of JMY and is translocated to nascent autophagosome membranes to promote WH2-dependent F-actin nucleation necessary for autophagosome formation and movement. JMY cooperates with Arp2/3 mediated F-actin assembly and branching, forming a comet-like F-actin network necessary for the movement of the autophagosome [82]. This activity of JMY is regulated by STRAP, which interacts with JMY in a fed state or basal autophagy to suppress its translocation to nascent autophagosome, actin nucleation activity, and LC3 interaction. Genetic ablation of STRAP or starvation reverses this suppression. These findings show that STRAP acts as a negative regulator of autophagy by inhibiting JMY and preventing its recruitment and actin-nucleation function during autophagosome expansion [82].

Coronin 1B (Coro1B) is an actin binding protein that regulates actin remodeling to maintain cell shape and internal structure and promotes cell migration via formation of lamellipodia at the leading edge [83], as well as maintaining endothelial cell–cell junctions. Coro1B coordinates filament formation by inhibiting filament nucleation by Arp2/3 complex, which mediates actin branching. It also regulates actin severing by recruiting SSH1L to dephosphorylate and activate Cofilin’s severing activity. These functions allow Coro1B to remodel actin turnover [83]. Coro1B negatively regulates autophagy by restricting remodeling of peri-lysosomal F-actin structures. This in turn restricts V-ATPase recruitment and suppresses lysosomal acidification [84]. Through this mechanism, Coro1B suppresses basal levels of autophagic flux. Loss of Coro1B or USP45, which stabilizes Coro1B by deubiquitylation, releases the restriction of peri-lysosomal actin remodeling, promoting constitutive V-ATPase recruitment to lysosomes and enhanced excessive lysosomal acidification. Consequently, excessive autophagic flux results from the loss of these proteins, highlighting the role of Coro1B in autophagic flux regulation [84].

F-actin capping proteins or stabilizing proteins may also negatively regulate autophagy. CapZ binds to fast-growing ends of actin filaments to inhibit elongation, which is important for cell movement, structure, and morphology [85]. CapZ is recruited to the phagophore site to cap F-actin barbed ends, promoting localized actin assembly within the growing phagophores to maintain its curvature, thereby controlling autophagic flux. Loss of CapZ subunit β reverses the controlled actin assembly, enhancing phagophore remodeling, abnormal phagophore morphology, and defective autophagosome formation [86]. These findings suggest that the coordinated regulation of actin dynamics by CapZβ is essential for shaping developing phagophore and autophagosome maturation.

All these findings point to a paradigm where autophagic flux can be enhanced or inhibited by targeting actin binding proteins that regulate actin dynamics.

## 3. Autophagy–Actin Crosstalk in Cellular Homeostasis

**Figure 4:**
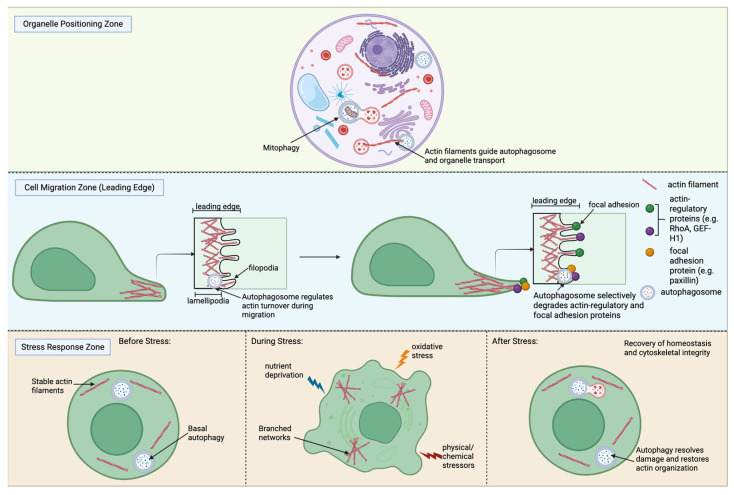
Integrative model of autophagy–actin crosstalk in maintaining cellular homeostasis as it applies to migration, stress, and organelle positioning. Created in BioRender. Fierti, A. (2026) https://BioRender.com/j1z3c6v accessed on 12 February 2026.

Autophagy and actin dynamics converge to sustain the internal homeostasis of eukaryotic cells. The actin cytoskeleton provides structural support and drives cellular movement, while autophagy maintains quality control by recycling damaged or obsolete components [59,87]. Their crosstalk coordinates key processes including cell shape, organelle positioning, migration, adhesion, and stress adaptation [88] as shown in Figure 4.

### 3.1. Relevance for Maintenance of Cell Shape and Organelle Positioning

The actin cytoskeleton is a primary determinant of cell shape; the dynamic assembly and disassembly of actin filaments is what is required for cells to move and change shape [89]. The cytoskeleton forms a dynamic scaffold of filaments throughout the cytoplasm that supports the structure of the plasma membrane and promotes intracellular organization [90]. Cortical actin provides structural support, preserves membrane integrity, and mediates interactions between neighboring cells [91]. Stress fibers that span the cytoplasm generate tension and help maintain overall cellular architecture [91]. Actin filaments also form localized networks around specific organelles, such as the nucleus or mitochondria, to facilitate their proper positioning and intracellular transport [59]. Autophagy aids these processes by selectively removing degraded organelles and protein aggregates to ensure that cytoskeletal organization is not impacted by a buildup of cellular debris [88]. For example, mitophagy is a specialized form of autophagy that mediates and selectively degrades damaged mitochondria, supporting mitochondrial quality control and cellular homeostasis [92]. The crosstalk between actin and autophagy allows cells to maintain structural integrity and optimize organelle distribution, which is critical for efficient cellular function and adaptability [59].

### 3.2. Role in Cell Migration and Adhesion

Cell migration is a fundamental process in cellular development, wound healing, and cancer metastasis [93]. Efficient migration depends on the coordinated polymerization and depolymerization of actin filaments, which organize into protrusive structures at the leading edge, such as lamellipodia and filopodia, and contractile structures, including stress fibers, that generate tension to pull the cell body forward and retract the rear [87]. These actin structures are closely linked to adhesion complexes, consisting of focal complexes and focal adhesions, which anchor the cell to the extracellular matrix and transmit forces generated by the cytoskeleton [94]. Proper cellular migration requires a balance between protrusive and contractile forces, as overactive contractility can hinder movement while insufficient adhesion reduces traction, both of which decrease cellular motility [95]. At the molecular level, actin nucleators like the Arp2/3 complex and formins drive the formation of branched and linear filaments, respectively, while actin binding proteins, such as cofilin and cortactin, regulate filament turnover, enabling rapid cytoskeletal remodeling in response to environmental cues [96].

Autophagy supports the work of these dynamic actin structures by regulating the availability and turnover of actin-remodeling proteins, thereby influencing the dynamics of protrusions, stress fibers, and adhesion structures [44]. Two key regulators of cytoskeletal tension and cell migration are RhoA, a small GTPase controlling stress fiber formation and contractility, and GEF-H1, a guanine nucleotide exchange factor that activates RhoA and links microtubule dynamics to actin remodeling. Their coordinated activity balances protrusive forces at the leading edge with contractile forces at the rear, enabling directional migration. Selective autophagic degradation of RhoA and GEF-H1, mediated by p62-dependent pathways, fine-tunes this balance and optimizes actin filament dynamics for efficient cell movement [97,98]. Similarly, autophagy regulates focal adhesion turnover by degrading focal adhesion proteins such as paxillin, which are critical for efficient cell detachment and forward movement, and has been well characterized in migrating cancer cells [98,99]. Conversely, actin filaments provide a structural framework that guides where autophagosomes form and move, helping autophagy occur in the right locations within the migrating cell [60]. Through this reciprocal regulation, autophagy–actin crosstalk ensures that cells maintain both the structural integrity and adaptability needed for directional migration and dynamic adhesion to the extracellular environment. Dysregulation of this interplay has been implicated in impaired wound healing, defective immune cell trafficking, and increased metastatic potential in cancer [61,62,63].

### 3.3. Autophagy and Actin Balance in Stress Response and Adaptation

Eukaryotic cells constantly encounter fluctuating environmental conditions, including changes in nutrient availability, oxygen tension, redox state, and exposure to physical or chemical stressors [100]. When these fluctuations surpass a certain threshold, they are perceived as cellular stress, triggering adaptive responses that determine whether the cell survives or undergoes damage [101]. Among these responses, autophagy plays a key role in maintaining cellular homeostasis by sequestering damaged organelles and proteins via autophagosomes that fuse with lysosomes for degradation and recycling. This process allows cells to maintain energy balance and metabolic adaptation during stress, serving as a protective mechanism against degenerative, inflammatory, and infectious challenges [98].

The actin cytoskeleton functions alongside autophagy as a dynamic structural framework that supports stress adaptation. Under stressful conditions, actin filaments are reorganized to facilitate the trafficking of damaged organelles and protein aggregates toward autophagic compartments to enable efficient degradation [87]. Actin-dependent processes, such as membrane remodeling and vesicular transport, are critical for autophagosome formation, trafficking, and fusion with lysosomes, highlighting the interdependence of autophagy and actin dynamics. This crosstalk ensures that cells can adapt their shape, organelle positioning, and intracellular transport in response to stress, maintaining structural integrity and homeostasis [98].

Autophagy and the actin cytoskeleton operate as integrated components of the cellular stress response, coordinating catabolic and structural pathways to preserve cell function under adverse conditions [59]. Understanding this interplay provides insight into how cells respond to environmental challenges and highlights potential therapeutic targets for diseases associated with stress response dysregulation [20].

Together, these pathways illustrate that autophagy and actin function as an integrated network maintaining cellular homeostasis. Through continuous feedback between cytoskeletal remodeling and degradative recycling, cells preserve structural organization, adaptability, and survival under both steady-state and stress conditions.

## 4. Implications for Cell Death and Disease

### 4.1. Autophagy–Actin Interactions in Apoptosis

Building on the role of actin–autophagy interactions in stress adaptation, dysregulation of these pathways contributes to cell death and disease. Under conditions of excessive or prolonged stress, the accumulation of damaged proteins and organelles can drive disease progression. In neurodegeneration, this buildup causes toxicity and cell death, whereas in cancer, it promotes genomic instability, altered metabolism, and survival advantages that facilitate tumor growth [20]. Disruption of actin–autophagy balance impairs stress adaptation, compromising cell survival and promoting disease [102].

Actin dynamics are involved in the regulation of apoptotic signaling. Because of the close relationship between the failure to properly carry out apoptosis and tumor malignancy, tumor cell transformation is theorized to be aided by actin cytoskeletal remodeling that evades normal apoptotic signaling [103]. Actin links nutritional sensing and oxidative damage to initiation of apoptosis in a mitochondrial-dependent manner [104]. Cytoskeletal components are capable of both initiating and inhibiting apoptosis in highly conserved mechanisms; however, anucleate cells such as platelets lack actin-dependent apoptosis and apoptotic markers are unaffected by Cytochalasin D or actin polymerizing drugs [105].

During apoptosis, F-actin is cleaved to facilitate disintegration of the cell. Actin depolymerization by Cytochalasin D and actin filament aggregation by jasplakinolide can also induce apoptosis, although the two mechanisms display different morphology of focal adhesion, protein expression, and pro-caspase-8 cleavage. However, both mechanisms form an apoptotic death-inducing signaling complex, indicating actin’s role in apoptotic signaling [106]. Piezo1, a gene encoding part of a mechanosensitive ion channel, also links F-actin to apoptosis in human anterior vaginal wall fibroblasts in response to mechanical stress [107].

Gelsolin, an actin regulated protein, regulates cell motility and growth by modulating the exchange between G-actin and F-actin. Its overexpression inhibits apoptotic signaling in Jurkat cells; however, this is done by suppressing CPP32 protease activity, rather than by altering F-actin morphology [108]. Advillin, a protein in the gelsolin superfamily, is involved in filament nucleation, actin bundling and disassembly, and actin capping. It regulates actin dynamics by facilitating polymerization and depolymerization, and thus plays a potent role in cancer growth and metastasis by promoting uncontrolled tumor cell growth [49,109].

Autophagy directly modulates apoptotic signaling. It promotes cathepsin D maturation and localization, which then induces apoptosis by activating caspase 3 [110]. Cucurbitacin B inhibits malignant proliferation in Jurkat cells by disrupting actin dynamics and inducing autophagy. In cells with suppressed autophagy, cucurbitacin B also promotes apoptosis via increased caspase-3 expression [111].

### 4.2. Dysregulation in Disease Context

Autophagosomes, formed by autophagy capture, degrade cellular components and recycle these components to provide nutrients to other parts of the cell. Cellular stressors including organelle damage and abnormal protein presence trigger this mechanism. Basal autophagy levels suppress tumor formation early in tumorigenesis. However, autophagy promotes progression later in tumor development by providing necessary nutrients for growth and facilitating metastasis [112]. Tumors can upregulate autophagy to increase nutrient supply. In addition to providing a steady supply of nutrients, autophagy suppresses p53 induction and regulates mitochondrial function, promoting survival in tumor cells [61]. In fact, some cancers depend on the autophagic suppression of therapeutic agents for survival [113]. For example, pancreatic primary tumors require increased basal autophagic levels, such that therapeutic inhibition of autophagy damages the tumor cells and suppresses growth in vitro [114]. On the other hand, prolonged autophagy can lead to autophagic cell death, causing the self-destruction of tumor cells via uncontrolled degradation of cellular components [115]. Loss of autophagy-related genes may allow more aggressive development of HER2+ breast cancer. However, increased autophagy increases tumorigenesis by inducing interactions between Beclin 1 and HER2 [116]. Overexpression of the CLDN6 breast cancer suppressor gene inhibits tumor metastasis by regulating autophagy via positive feedback of WIP expression, which induces WIP-dependent F-actin assembly [117]. Inhibition of autophagy is becoming a promising target for cancer therapies and repurposing of drugs such as hydroxychloroquine, a medication for malaria and autoimmune conditions that inhibits autophagy by increasing the pH of autophagolysosomes and preventing degradation of cellular components [118].

Autophagy serves as a mechanism for combating the effects of neurodegenerative diseases that lead to dysfunctional protein aggregation in neurons and promises potential therapeutic benefits [119]. Neuronal cells depend significantly on autophagic mechanisms to clear dysfunctional proteins and organelles because post-mitotic neurons are unable to independently remove these unwanted aggregates [120]. In fact, many proteins associated with neurodegeneration are substrates in autophagy [121]. Specifically, amyloid-beta and tau protein accumulations in Alzheimer’s disease increase when autophagy is dysfunctional [122]. Histone deacetylase-6 (HDAC6) modulates autophagolysosome formation in selective autophagy by recruiting actin remodeling machinery. This form of autophagy targets neurodegenerative protein aggregates and damaged mitochondria [123]. Autophagy declines in an age-related manner, which may be implicated in the age-associated increase in neurodegenerative diseases. Autophagy decline decreases the ability to maintain neuronal homeostasis and prevent accumulation of toxic protein aggregates [124]. This decline is in part due to inhibition by neurodegeneration-associated proteins, which accumulate as a result of autophagy decline [121]. Protein homeostasis is significantly maintained by autophagy; massive neuronal loss occurs in autophagy-deficient mice [125]. In microglia, autophagic dysregulation affects inflammation and the ability to carry out phagocytosis of apoptotic cells and dysfunctional protein aggregates. Autophagy regulation is linked to the microglial inflammatory phenotype, which promotes age-related neuronal dysfunction [126]. Mutations in the WDR45 gene are thought to cause β-propeller protein-associated neurodegeneration (BPAN) via dysfunction of WIPI β-propeller proteins, which act as scaffolds during autophagy. This impairment of autophagy contributes to neurodegeneration and neuronal iron accumulation, pathological symptoms of BPAN [127]. However, the literature has not reached a consensus about the mechanism through which WDR45 mutations cause BPAN, as WIPI4 depletion has also been shown to cause autophagy-independent ferroptosis [128].

Autophagy is involved in the cellular response to pathogens. The Wnt5A-Rac1-Disheveled-mediated actin-associated autophagy circuit is a part of the innate immune system in macrophages, as it helps degrade bacterial pathogens [129]. The actin cytoskeleton supports autophagosome formation and development, which help ensure homeostasis in response to bacterial and viral threats [73]. Atg9B regulates bacterial internalization by controlling actin dynamics; its inhibition is necessary for bacterial cell internalization [130]. As a result of actin’s role in supporting autophagy, many pathogens target the actin cytoskeleton to inhibit autophagy and other membrane remodeling processes [131]. Bacterial pathogens alter actin–plasma membrane interactions to facilitate their entry into the cell. Rickettsia parkeri uses outer membrane protein B to block bacterial surface protein ubiquitylation, which enables the bacteria to evade autophagy [132]. While pathogens must evade autophagic degradation, many also exploit the pathway to replicate and infect other cells. Mycobacterium tuberculosis-infected vacuoles are induced by autophagy to eject from the host cell [132]. However, other pathogens can replicate within the autophagosome and avoid the cell’s attempt to degrade and eject the pathogen. Porphyromonas gingivalis depends on this mechanism to avoid degradation within host cells. In fact, therapeutic induction of autophagy can increase pathogenic growth and decrease degradation of the pathogens [133]. Several pathogens also take advantage of the actin cytoskeleton itself to infect other cells. Shigella recruits septin GTP-binding proteins to polymerize actin within the cytosol to spread between cells via septin cages [134].

## 5. Therapeutic Implications

### 5.1. Cancer

Regulation of autophagy is an increasingly promising mechanism for therapies to improve cancer, neurodegeneration, and infectious disease prognoses. The tumor suppressor CLDN6 has already been identified as a potential target for breast cancer therapies, as its overexpression inhibits metastasis in preexisting tumors via positive autophagic regulation. CLDN6 induces autophagy by increasing autophagosome synthesis, as indicated by increases in cellular LC3II/I, Atg5, and Atg7 levels [117]. This effect could be used to detect breast cancer and limit tumor growth by upregulating CLDN6 in individuals with preexisting breast cancer [117]. Both negative and positive regulation of autophagy have implications for cancer therapy, both as prevention measures and treatments to limit tumor growth. By downregulating autophagic pathways in cancer cells, it may be possible to starve these tumors by preventing them from acquiring necessary levels of nutrients or metastasizing to other tissues. On the other hand, upregulation of autophagy pathways may repress tumorigenesis. The therapeutic potential of both positive and negative regulation of autophagy pathways suggests the possibility of multiple avenues of treatment, thus providing more options for patients in need of novel cancer therapies.

The *AVIL* oncogene, which encodes an actin binding protein Advilin (AVIL), regulates actin dynamics and has been shown to be a viable target of glioblastoma therapies [135,136]. Advilin plays a role in cell migration by promoting the growth of filopodium-like structures. Its involvement in facilitating cell growth and migration results in an oncogenic effect that allows tumors to grow more effectively, as seen in temozolomide-resistant glioblastoma tumors. Advilin is overexpressed in glioblastoma, and its silencing kills glioblastoma cells in vitro, with significant inhibiting effects in vivo [109]. *AVIL* also forms the *MARS-AVIL* RNA fusion, which leads to rhabdomyosarcoma tumorigenesis. Silencing of the gene fusion results in similar destruction in vitro and inhibition in vivo of rhabdomyosarcoma cells as is seen in glioblastoma [137]. AVIL mutants with disrupted actin binding exhibit lower tumorigenesis, as well as decreased tumor volume and weight. Additionally, direct competitive inhibition of AVIL by Compound A selectively prevents interactions with actin in tumor cells [135]. Compound A has strong implications for glioblastoma therapies, as it effectively targets glioblastoma cells without negative side effects in a murine model. However, Compound A must be further optimized and studied to maximize its benefit as a cancer therapy.

### 5.2. Neurodegeneration

Age-related decline in autophagic activity is strongly associated with onset of neurodegeneration, indicating that positively regulating autophagy may be beneficial for maintaining cognitive function in the elderly and treating progressive neurodegenerative diseases. Upregulation of QC autophagy may be used to clear pathological accumulations within the brain, such as those of amyloid-beta and tau [74].

### 5.3. Infectious Disease Progression

Autophagy’s role in regulating inflammation and infectious disease infection may provide mechanisms to mitigate such diseases. However, because autophagy can be both beneficial and harmful to various pathogens, therapies must be developed with precise understanding of the pathogens’ mechanisms of action, in order to avoid exacerbating the disease. This is the case for cancer therapies as well, given that autophagy inhibition may effectively limit tumor growth and metastasis but may also allow greater tumorigenesis. Antiviral therapies must selectively target autophagy with specific degradative effects to effectively combat infection without enhancing the pathogens’ infectious ability [138]. Current antiviral therapies that regulate autophagy include metformin, which increases insulin sensitivity and autophagy and may be an effective therapy against SARS-CoV-2, wortmannin, which inhibits autophagy through P13K and limits ZIKV replication, and ivermectin, which induces autophagy and is also related to SARS-CoV-2 propagation. Ivermectin in particular induces autophagy by inhibiting AKT phosphorylation, which influences F-actin stability [138]. Evidently, both the induction and inhibition of autophagy may be beneficial when developing new antiviral therapies or repurposing existing therapies. These therapies must be developed with a clear knowledge of how the target pathogen utilizes or evades autophagy in order to best target the disease.

## 6. Conclusions and Future Perspective

Actin, a key component of the cytoskeleton, plays diverse roles in cellular processes. Autophagy plays an essential role in cells, regulating homeostasis by recycling nutrients and degrading unnecessary cellular components. Autophagy and actin polymerization dynamics are deeply interconnected, from the actin-mediated formation of the phagophore, phagosome maturation, autophagolysosome to autophagy-regulated cell migration, and gamete generation. Various drugs that inhibit actin polymerization have been shown to limit autophagolysosome development by preventing the formation of actin structures that facilitate autophagosome–lysosome fusion. On the other hand, autophagy regulates actin turnover to regulate actin dynamics. This bidirectional interplay, supported by a growing body of evidence, is highly conserved, functions as an integrated system rather than in isolation, and is essential for cellular homeostasis.

Despite the significant advances to understand the actin–autophagy interplay, underlying molecular gaps remain elusive. It is unclear how spatial and temporal regulation of actin remodeling determine whether selective or non-selective autophagy occurs. While global reduction in actin remodeling (pharmacologically induced) may hinder autophagy [70], starvation triggers global depolymerization of F-actin, which allows for localized polymerization for proper formation and maturation of the autophagosome [86]. However, if starvation-induced depolymerization is occurring, how does that support the formation of actin comet on autophagosomes? Furthermore, how does this actin remodeling facilitate V-ATPase translocation to lysosome, a process that is dependent on actin remodeling? The mechanism through which global reduction in actin remodeling promotes autophagic flux remains elusive.

Targeting autophagy has promising implications for cancer therapies, neurodegenerative disease prevention and treatment, and inflammatory and infectious disease mitigation. A number of therapeutic strategies have already been developed to target autophagy through both inhibition and activation [139]. While significant strides are already being made in the realm of cancer therapies, more research needs to be done to strengthen these possible treatments and expand their applications to other diseases.

## Data Availability

No new data were created or analyzed in this study. Data sharing is not applicable to this article.

## Abbreviations

The following abbreviations are used in this manuscript:

ABPs	Actin binding proteins
AMPK	AMP-activated protein kinase
Arp2/3 complex	Actin-related protein 2/3 complex
Arps/ARPs	Actin-related proteins
Atg/ATG	Autophagy related gene/protein
ATG14L	Autophagy-related protein 14-like
AVIL	Oncogene which encodes actin binding protein Advilin
BPAN	β-Propeller protein-associated neurodegeneration
CapZ	Capping actin protein of muscle Z-line
Cav-1	Caveolin-1 protein
CLDN6	Claudin-6
CMA	Chaperone-mediated autophagy
Coro1B	Coronin 1B
ER	Endoplasmic reticulum
F-actin	Filamentous actin
FIP200	Focal adhesion kinase-interacting protein of 200 kDa
G-actin	Globular actin
HDAC6	Histone deacetylase 6
HER2	Human Epidermal Growth Factor Receptor 2
HOPS complex	Homotypic Fusion and Protein Sorting complex
Hsc70	Heat Shock Cognate 70 kDa protein
INO80	INO80 chromatin-remodeling complex ATPase subunit
JMY	Junction mediating and regulatory protein
KFERQ-like motif	Pentapeptide amino acid sequence (K-F-E-R-Q) that targets proteins for chaperone-mediated autophagy
LAMP-2A	Lysosome-associated membrane protein 2A
LC3	Microtubule-associated protein 1A/1B light chain 3
LIR	LC3-interaction region
mTORC1	Mammalian Target of Rapamycin Complex 1
PI3KC3-C1	Class III Phosphatidylinositol 3-kinase Complex I
PI3P	Phosphatidylinositol 3-phosphate
QC	Quality control
Rab7	Rab GTPase 7
SNARE	Soluble N-ethylmaleimide–sensitive factor attachment protein receptor
SSH1L	Slingshot 1-L
STRAP	Serine–Threonine Kinase Receptor-Associated Protein
ULK1	Unc-51-like autophagy activating kinase 1
USP45	Ubiquitin carboxyl-terminal hydrolase 45
VPS15	Vacuolar protein sorting 15
VPS34	Phosphatidylinositol 3-kinase catalytic subunit type 3
WASH	Wiskott–Aldrich syndrome protein and SCAR homologue
WASP	Wiskott–Aldrich syndrome protein
WDR45	WD repeat domain 45 gene that encodes the WIPI4 protein
WH2	WASP homology 2
WIP	Wiskott–Aldrich syndrome protein–interacting protein
WIPI4	Protein encoded by the WDR45 gene
